# Digital consent in gynecology: an evaluation of patient experience

**DOI:** 10.1007/s00404-023-07304-1

**Published:** 2023-12-08

**Authors:** Laura Burney Ellis, Jennifer Barcroft, Edward St John, Dafydd Loughran, Maria Kyrgiou, David Phelps

**Affiliations:** 1 Imperial College London, Imperial College Healthcare NHS Trust, IRDB, Du Cane Road, London, W12 0NN UK; 2 Portsmouth Hospitals University NHS Trust, Cosham, Portsmouth, PO6 3LY UK; 3 University of Portsmouth, University House, Winston Churchill Ave, Portsmouth, PO1 2UP UK; 4 Concentric Health, Tramshed Tech, Cardiff, CF11 6BH UK; 5 University Hospitals Southampton NHS Foundation Trust, Princess Anne Hospital, Coxford Road, Southampton, SO16 5YA UK

**Keywords:** Electronic consent, Digital consent, e-Consent, Patient experience

## Abstract

**Introduction:**

The surgical consent process is a crucial discussion between patient and surgeon, which is predominantly documented utilizing hand-written forms. The exchange of individualized information allows the patient to make a truly informed decision. Digital consent (also known as electronic consent or e-consent) has been shown to improve accuracy of information provided without increasing the time taken to consent patients. We aimed to evaluate patient experience and effectiveness of digital consent in a gynecology department in a tertiary London Teaching Hospital.

**Methods:**

A questionnaire was designed and completed by 100 patients undergoing gynecological surgery: 50 consented using paper and 50 consented digitally. The questionnaire included 8 statements, with five possible answers to select, ranging from strongly agree to strongly disagree, on a standard five-point Likert Scale. Patients were all female and categorized into age groups (deciles) and asked whether consent was taken digitally or on paper. Data were collected between January and July 2021.

**Results:**

Most responses were positive with 87% (694/800) of responses to the questions being either strongly agree or agree. Patients who were consented using paper selected ‘strongly agree’ 43.5% (174/400) of the time in comparison to 64.8% (259/400) of the time when they were consented digitally. The majority, 86% (43/50), of digitally consented patients received a copy of the consent form in comparison to 18% (9/50) of those consented using paper. On average, the patients consented digitally were older than their paper-consented counterparts (49–58 and 59–68 respectively). The mean scores for the questions relating to the ease of reading the form, ease of understanding the form, understanding of the potential complications, and overall satisfaction were higher in those digitally consented (*p* < 0.05).

**Discussion:**

Overall, patients were satisfied with both methods of consent. However, individuals who were consented digitally reported higher levels of satisfaction throughout the consent process, compared to paper consent. These data suggest that digital consent is an acceptable alternative to paper consent for patients and facilitates adherence to national consent guidance, which stipulates patients should be given the information they request.

## What does this study add to the clinical work

**Table:** 

Digital consent appears to be acceptable to patients undergoing gynecological surgery. Mean scores were higher for the majority of questions in a Likert scale questionnaire, and this was statistically significant for questions regarding ease of reading and understanding, understanding of complications, and overall satisfaction.	

## Introduction

Consenting to an operation is a pivotal moment in a patient’s journey. Guidance regarding consent from the Royal College of Surgeons, England (RCS Eng), states that consent ‘… is not merely the signing of a form. It is the process of providing the information that enables the patient to make a decision to undergo a specific treatment’ [1]. It must be provided in a way that each patient can comprehend. The Royal College of Obstetricians and Gynaecologists (RCOG) Clinical Governance Advice no. 6 recommends that doctors must ensure ‘that she is fully informed, understands… the risks of receiving no treatment, as well as any reasonable or accepted alternative treatments…The risks of the proposed procedure and the likelihood of complications should be presented in a fashion that the patient is able to understand’ [2]. Guidelines across specialties agree that the consent process should be a meaningful, detailed two-way discussion [3], with the focus being on provision of information tailored to each individual patient [3, 4].

Currently, in the UK, the consent discussion is typically documented using a paper-based, carbon copy form, adapted from a template provided by the Department of Health, updated in 2009 [5], which has pre-filled headings with a blank space beneath for the clinician to handwrite the details. Paper consent forms have the benefit of being entirely customizable to each individual patient, but nevertheless have some disadvantages. First, they are dependent on the consent-taking healthcare professional to accurately and comprehensively recall and handwrite the risks [6]. Paper forms are therefore more vulnerable to errors, omissions, and illegibility [7–9]; ‘core risks’ were omitted on over 90% of paper consent forms in a recent study in a general surgical department in a UK-based trust [10]. Second, they contain a finite amount of space for documentation, and there is often insufficient space to expand on these risks in full, use lay explanations, or to offer any written detail about the likelihood of these risks occurring, as recommended by surgical Royal Colleges (RCS, RCOG [1, 2]). There is not enough space for risks to be presented in any way other than a long, sequential, comma separated list, which can be overwhelming for patients, lacking in context, and difficult to retain [11].

Additionally, a carbon copy form is more likely to be illegible [6] or mislaid [12], can only be read by one person at one time [13], and in practice giving a copy to the patient is often forgotten [14]. Paper-based forms can also result in delays, demonstrated by a study in California, USA, which identified missing/incomplete consent as the most common reason for surgical delay [15]. Handwritten forms rely upon each healthcare professional’s judgment about which risks to include; in a UK-based study, 77% of surgical trainees said they would strongly support an online consenting resource [16].

*Concentric* [17] is a digital consent online platform that enables the creation of tailor-made surgical consent forms. Risks are automatically pre-populated, but can be selected, rejected, or refined by the consenting clinician, supporting individual personalization. Risks are presented in digestible and clinically relevant sections: ‘immediate’, ‘early’, and ‘late’. The categories of risks include: ‘common’: > 1/20, ‘less common’; < 1/20, and ‘rare’: < 1/100. Risks can be expanded, providing a detailed lay explanation, for example, explaining scenarios when a specific risk may be more likely to occur, the reasons they might happen or consequences of the complications, so each patient can understand more comprehensively which risks may be individually relevant to their specific case. The consent form can either be shared with the patient remotely or face to face, enabling the sharing of consent information in advance, giving the patient time to consider the proposed procedure, as well in person discussion. The interactive online format allows the patient and their health professional to ‘click through’ the content including the risks, and the patient can sign electronically. The form is automatically uploaded to each hospital’s individual online patient record. A copy of the form is shared via text message or email to the patient, along with any included hospital or department-specific information leaflets. As *Concentric* runs as a web app, it can be opened on any device connected to the internet, making it readily available, portable and interoperable with other digital health systems.

Preliminary research, in specialties outside gynecology, has shown that use of digital consent can reduce errors and omissions [6, 10, 15]. Use of electronic resources in surgical consent has been shown to improve patient’s knowledge of the procedure [18]. Digital consent has been shown to be equivalent to paper consenting in the time taken to consent [19], and may even be faster [20, 21]. In 2020, a systematic review of the use of digital consent in research concluded that in comparison to paper forms, digital consent has the potential to improve the informed consent process overall [22].

Although digital or electronic consent has many tangible benefits over paper consent, there is a need to evaluate its use from a patient experience point of view, particularly in consenting for Gynecological procedures, which often necessitate sensitive discussions, such as the impact on future fertility [23]. We aimed to assess the patient’s experience of digital consent in a Gynecological setting, at a Tertiary London Teaching Hospital.

## Methods

Trust service evaluation approval (Registration number GRM_076) was granted for all phases of the project, and ethical approval was not required.

**Fig. 1:**
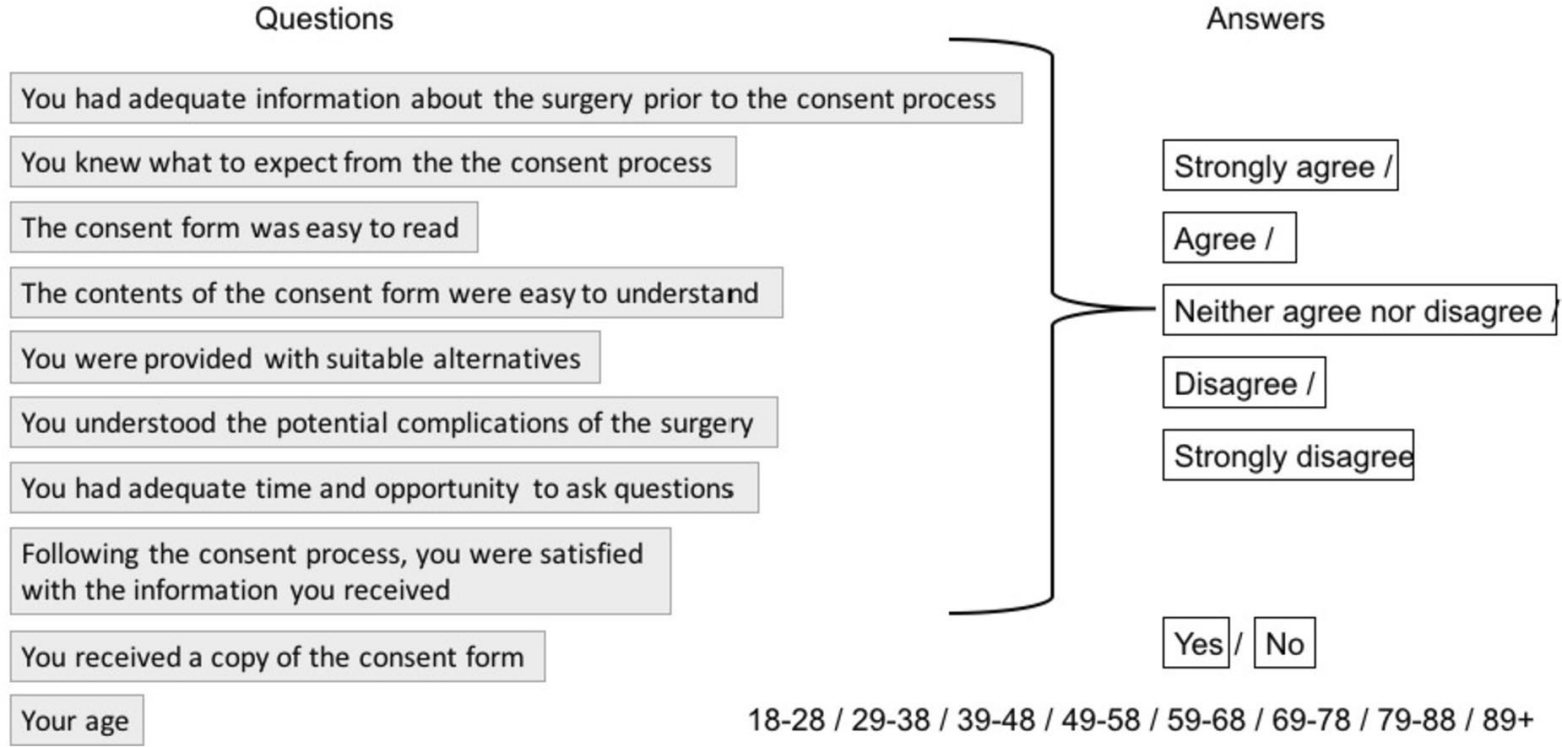
Patient questionnaire, outlining specific questions relating to consent and their corresponding multi-choice answers

A questionnaire evaluating patient experience was created. Ten questions were included. Nine were phrased as statements. A 5-point Likert scale [24] was used to be able to quantify responses for eight of the 10 questions; answers were as follows: strongly agree, agree, neither agree nor disagree, disagree and strongly disagree. One question had a binary ‘yes’ or ‘no’ response. One question was included on the patient’s age. Questions were designed by the first author and edited and finalized by the senior author. The included questions are detailed in Fig. 1. The questionnaire responses were anonymized (Tables 1, 2, 3).

Patients were selected at random from elective gynecological or gynecology–oncology theater lists. One hundred and twelve eligible patients were approached after their surgery and asked if they would like to take part in the study; 12 patients declined (seven who had been consented using paper and five who had been digitally consented). The study was closed once 100 patients undergoing gynecological surgery at our center agreed to take part in the study (50 patients who had been consented using paper and 50 patients who had been digitally consented using *Concentric).* All participants completed all ten questions on the questionnaire and were included in the study. All patients underwent surgery between January 2021 and July 2021 and took part in the study within a minimum of 6 h and a maximum of 7 days from the time they were consented for surgery. All gynecological operations were included in the study, which included day case operations, such as hysteroscopies, laparoscopic surgeries, and major abdominal surgery, such as primary debulking surgery. The questionnaire was distributed and collected by a junior doctor who was not involved in any of the participating individuals’ consent. Patients were consented by the usual clinician who was responsible for their operation; the total number of consenting clinicians was 11, including both registrars and consultants. All clinicians consented using both methods during the study period. Patients were informed that the results would be anonymous and that no answers would be directly feedback to their lead clinician or their team.

Mean scores for each group were calculated (Strongly disagree = 1, disagree = 2, neither agree nor disagree = 3, agree = 4, strongly agree = 5). Independent *t*-tests were conducted for each Likert Scale question.

Data were processed and analyzed using Microsoft Excel [25], and statistical analysis was conducted using R studio [26] (*tidyverse* package [27]).

## Results

**Fig. 2:**
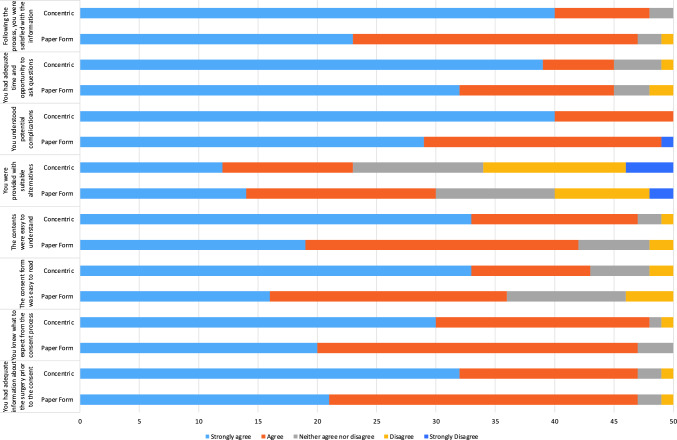
Bar chart displaying results for the Likert scale questions

In both groups, 94% (47/50) agreed or strongly agreed that they had received adequate information prior to the consent process. However, digitally consented patients were 1.5 times more likely to ‘strongly agree’ (mean scores 4.34 [paper-consented] and 4.56 [digitally consented]). 94% (47/50) of paper-consented and 96% (48/50) of digitally consented agreed or strongly agreed that they knew what to expect from the consent process (mean scores 4.34 and 4.54) (Tables 1, 2, Fig. 2)

**Table 1:** Likert scale question results for both groups

		Strongly agree	Agree	Neither agree nor disagree	Disagree	Strongly disagree
You had adequate information about the surgery prior to the consent	Paper form	21	26	2	1	0
	Concentric	32	15	2	1	0
You knew what to expect from the consent process	Paper form	20	27	3	0	0
	Concentric	30	18	1	1	0
The consent form was easy to read	Paper form	16	20	10	4	0
	Concentric	33	10	5	2	0
The contents were easy to understand	Paper form	19	23	6	2	0
	Concentric	33	14	2	1	0
You were provided with suitable alternatives	Paper form	14	16	10	8	2
	Concentric	12	11	11	12	4
You understood potential complications	Paper form	29	20	0	0	1
	Concentric	40	10	0	0	0
You had adequate time and opportunity to ask questions	Paper form	32	13	3	2	0
	Concentric	39	6	4	1	0
Following the process, you were satisfied with the information	Paper form	23	24	2	1	0
	Concentric	40	8	2	0	0

**Table 2:** Likert Scale Questions - Mean scores and statistically significant results

Age bracket	18–28	29–38	39–48	49–58	59–68	69–78	79–88	89 +
Paper form group	5	12	7	12	7	4	2	1
Concentric group	0	4	5	13	13	9	6	0

Digitally consented patients were more than twice as likely (33/50 compared to 16/50) to ‘strongly agree’ that the consent form was easy to read. The mean score for this question was 3.96 for the paper-consented group, and 4.48 for the digitally consented group, and this difference was statistically significant (*p* = 0.0002236). 84% (42/50) of paper-consented patients and 94% (47/50) of digitally consented patients agreed or strongly agreed that the contents of the consent form were easy to understand. 67% (33/50) of digitally consented patients agreed strongly with this statement, in comparison to 38% (16/50) of paper-consented patients. The mean score for this question regarding the understanding of the form was 4.18 (paper-consented), and 4.58 (digitally consented), and this difference was statistically significant (*p* = 0.008048).

60% (30/50) of paper-consented and 46% (23/50) of digitally consented patients agreed or strongly agreed that they were provided with suitable alternatives (mean scores: 3.64 [paper-consented], 3.3 [digitally consented]). 20% (10/50) of paper-consented and 22% (11/50) of digitally consented patients had a neutral response to this statement; 20% (10/50) of paper-consented and 32% (16/50) of digitally consented patients disagreed or strongly disagreed with this statement.

98% (49/50) of paper-consented patients, and 100% (50/50) of those digitally consented agreed or strongly agreed that they understood the potential complications. 80% (40/50) of digitally consented patients opted for ‘strongly agree’, in comparison to 58% (29/50) of those paper-consented. The mean scores for this question were 4.52 for paper-consented, and 4.8 for digitally consented, and this difference was statistically significant (*p* = 0.01684). 90% (40/50) of patients in both groups were in agreement that they had adequate time and opportunity to ask questions, but patients in the digital consent group were 20% more likely (39/50 vs 32/50) to agree strongly with this statement (mean scores 4.5 and 4.66).

Overall, when asked whether they were satisfied with the consent process and the information they received, 94% (47/50) and 96% (48/50) of paper-consented and digitally consented patients agreed respectively. 80% (40/50) of digitally consented patients agreed strongly, in comparison to 46% (23/50) of paper-consented patients. Regarding overall satisfaction with the process, the mean scores were 4.38 and 4.76, and this difference was statistically significant (*p* = 0.001948).

**Fig. 3:**
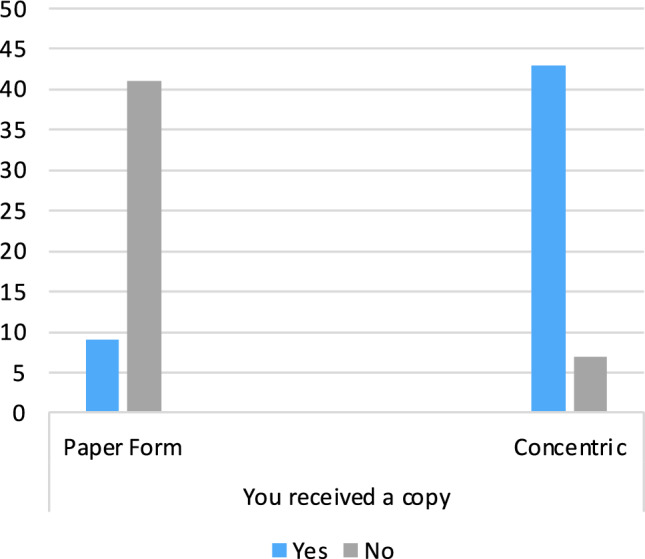
Bar chart displaying the distribution of patients who received copies of the consent form for both groups

86% (47/50) of digitally consented patients stated that they had received a copy of the consent form, in comparison to 18% (9/40) of paper-consented patients (Fig. 3).

The median age bracket was 49–58 for the paper-consented group and the median age bracket was 59–68 for the digitally-consented group. There was a higher proportion of those undergoing gynecological–oncological surgeries in the concentric group as compared to the paper consent group (42/50 vs 37/50) (Table 3).

**Table 3:** Age distribution for both groups

Question	Group	Score (mean)	*t* test	*p*-value	*p* < 0.05
You had adequate information about the surgery prior to the consent	Paper	4.34	1.6503	0.1021	
	Digital	4.56			
You knew what to expect from the consent process	Paper	4.34	1.6136	0.1098	
	Digital	4.54			
The consent form was easy to read	Paper	3.96	3.8331	0.0002236	*
	Digital	4.48			
The contents were easy to understand	Paper	4.18	2.7053	0.008048	*
	Digital	4.58			
You were provided with suitable alternatives	Paper	3.64	−1.374	0.1726	
	Digital	3.3			
You understood potential complications	Paper	4.52	2.4318	0.01684	*
	Digital	4.8			
You had adequate time and opportunity to ask questions	Paper	4.5	1.061	0.2913	
	Digital	4.66			
Following the process, you were satisfied with the information	Paper	4.38	3.1839	0.001948	*
	Digital	4.76			

## Discussion

Digitalization of healthcare is a rapidly evolving process. In 2019, in the UK, the NHS Long-Term Plan set a milestone for the use of digital technology in hospitals: by 2024, secondary care in England will be fully digitalised [28]. NHSX, now under the title of NHS England’s Transformation Directorate, encouraged NHS Trusts toward this goal with large monetary incentives under the heading of the Unified Tech Fund in 2021 [29]. NHS England’s Transformation Directorate’s most recent new framework is ‘The Year of the Digital Profession 2022’, ‘to support organizations to successfully deliver digital transformation’ [30]. Part of this mandate is to help equip hospitals with the appropriate tools to deliver efficient care and meet clinical demands; in a post-pandemic era with significant waiting lists for elective surgery not just in the UK but globally, it is vital that we truly streamline processes so we can meet these demands. As studies have shown digital consent to be faster than paper consent, as well as improving accuracy and reducing mislaid forms [10, 20], digital consent appears to be a useful tool in achieving these targets. To achieve patient-centred care, it is vital that technological innovations are appraised before implementation and consider patient experience as an essential part of the process.

Digital consent has been shown to be efficient, accurate, and acceptable to patients in many different healthcare settings including in research consent [31–33]. To our knowledge, this is the first study evaluating the use of digital consent in a gynecological setting and to assess the acceptability of digital to patients, in direct comparison to paper consent, in the real-world clinical setting.

Limitations of this study include the relatively small number of patients in each cohort, and the restrictiveness of the Likert Scale. By asking patients in the hospital if they would like to participate, there is likely to be an element of selection bias. However, as most patients agreed to participate (89%), this is unlikely to have skewed the results.

Our results indicate that overall, patients in our unit appeared satisfied with the informed consent process, whether consent was gained digitally or on paper. For seven out of the eight Likert Scale questions, the mean response was positive (either agree or strongly agree) for both digital and paper consent, and in the remaining question it was positive for paper consent and neutral for digital consent.

Although patients were satisfied with either method of consent, in our study, patients appear to be more satisfied with the consent process when consented digitally. In seven out of eight questions, patients consented using digital consent chose ‘strongly agree’ more often than those consented using paper. In five out of eight questions, digitally consented patients chose an overall positive response more often. The two questions with the highest discrimination between digital consent and paper were ‘the form was easy to read’ and ‘I understood the potential complications’. Being easy to read is a clear strength of digital consent. The text font is inevitably easier to read than a handwritten form. There is also the option to zoom in or have a ‘large text’ version for those with a visual impairment. When the form is sent digitally to the patient, they can open it on any device, improving the accessibility of the consent form. The digital form addresses issues with legibility of handwriting, which is consistently an issue among doctor’s in particular, supported by a study in Wales where doctors’ handwriting was statistically significantly worse when objectively analyzed by computer software [34]. A better understanding of the potential complications may be due to the supplementary information provided in the expandable sections; a randomized controlled trial of digital consent in pediatrics found that the supplementary material on the digital consent tool significantly improved understanding in a manner that did not negatively impact workflow [35], and a randomized trial of consent in research found comprehension improved with digital consent [21], although this finding was not statistically significant.

The only question where digital consent did not outperform paper consent was ‘I was provided with suitable alternatives’. We believe this may be due to the fact that our center is the tertiary referral center for gynecological cancers; it is likely that some patients felt that there were no other ‘suitable alternatives’ when the recommended treatment was a surgical option. There was a higher proportion of those undergoing gynecological–oncological surgeries in the concentric group as compared to the paper consent group. Previous studies evaluating digital consent in other specialties have found the contrary to be true [36, 37], and thus this result may be a reflection on the wording of this question, rather than on digital consent.

There are speculative concerns about the negative impact of the shift toward electronic documentation, including less eye contact with patients [38]. However, our results would suggest patients felt that they had more time and opportunity to ask questions when they were consented digitally. Although we did not collect data on the time taken to consent in our study, digital consent has been shown to be faster or time-equivalent to paper consent [21]. By reducing the time taken to handwrite or populate the form, it may facilitate more time for a meaningful dialog with the patient. This would infer that the consent process has potential to be more patient-specific when it is digital, as it may allow patients to have more time to ask about what is truly important to them as individuals. To further this, some digital consent tools are being developed that can adapt the material provided to each individual’s background [39]. This aspect contains significant possibilities for future development, including built-in translation software, audio options for visually-impaired patients, and even interactive videos to ensure comprehensive understanding.

The median age in the digitally consented cohort was over 60; as the responses in this cohort were mostly very positive, our study adds to current evidence suggesting that older patients are familiar with technology [40] and possibly do not experience any additional barriers to the general population [41].

A narrative review on digital consent [42] critiqued the provision of online information by stating that previous research suggested that online text is scanned rather than read [43]. However, these data were collected in 2005 using just 25 subjects and possibly is not reflective of electronic age we are now all part of.

Barriers to the implementation of digital consent include concerns about data security, legal framework replacing a paper version of consent [22], and clinician hesitancy for change [44]. Concentric is hosted on Google Cloud Platform that is fully compliant with all healthcare information governance requirements, with a data server within the UK [45]. In a Canadian study, 1 year after implementation of digital consent, a survey indicated an improvement in workflow and high overall satisfaction with the process [46]. Evidence from previous implementation suggests that although clinicians may be initially hesitant, they are satisfied with the process once they have experience using it.

Our study adds to the growing body of evidence suggesting that digital consent is likely to be superior to paper consent. Our study focuses on patient experience, which is essential in the delivery of ‘patient-centered care’. Overall, patients that were consented digitally had statistically significantly higher mean scores with questions relating to many aspects of consent process, including that the form was easy to read and understand, understanding of the potential complications and overall satisfaction. This study is the first to demonstrate the feasibility of digital consent in the context of gynecology. We believe that continued digitalization will ensure patients are fully informed and receive information in a manner acceptable to them: the surgical consent process may have found its place in the digital world.

## Data Availability

All available data is presented in this paper.
